# Plasma Cell-Free DNA Methylation-Based Prognosis in Metastatic Castrate-Resistant Prostate Cancer

**DOI:** 10.21203/rs.3.rs-6331572/v1

**Published:** 2025-04-21

**Authors:** Jodie Wong, Yijun Tian, Manishkumar S. Patel, Kapil Avasthi, Claire Hanson, Matt Larsen, Enos Ampaw, Muhammad Z.H. Fadlullah, Joseph Finklestein, Aik Choon Tan, Jong Park, Brandon J. Manley, Chiang-Ching Huang, Manish Kohli, Liang Wang

**Affiliations:** H. Lee Moffitt Cancer Center; H. Lee Moffitt Cancer Center; H. Lee Moffitt Cancer Center; H. Lee Moffitt Cancer Center; University of Utah, Huntsman Cancer Institute; University of Utah, Huntsman Cancer Institute; University of Utah, Huntsman Cancer Institute; University of Utah, Huntsman Cancer Institute; University of Utah, Huntsman Cancer Institute; University of Utah, Huntsman Cancer Institute; H. Lee Moffitt Cancer Center; H. Lee Moffitt Cancer Center; University of Wisconsin; University of Utah, Huntsman Cancer Institute; H. Lee Moffitt Cancer Center

## Abstract

Molecular prognostication in metastatic castration prostate cancer (mCRPC) remains challenging due to the lack of validated biomarkers. This study developed a plasma cell-free DNA (cfDNA) methylation-based prognostic model in mCRPC. Targeted cfDNA methylation sequencing in 96 prostate cancer patients in different states of cancer progression revealed 78 methylation haplotype blocks (MHBs) differentially methylated from organ-confined prostate cancer to mCRPC states. Among these 78 MHBs, the top 20 MHBs were associated with mCRPC overall survival and most MHB methylation levels positively correlated with predicted circulating tumor DNA (ctDNA) fraction. By integrating the MHB-based risk score with currently available prognostic clinical variables and ctDNA fraction a prognostic nomogram was developed which showed high predictive performance for mCRPC survival (AUC = 0.99 for 6 months, AUC = 0.90 for 1 year, and AUC = 0.87 for 2 years). These findings demonstrate potential of cfDNA methylation as a molecular biology-driven biomarker for mCRPC prognosis.

## Introduction

Prostate cancer is the second most common cancer in men globally and a leading cause of cancer-related deaths, with over 396,000 deaths annually worldwide^1^. Despite significant advancements in therapeutic strategies, survival for metastatic castration-resistant prostate cancer remains poor, with a median survival time of 30 to 34 months^2^, underscoring the need for improved prognostication methods. Since 2015, the management of metastatic hormone-sensitive prostate cancer (mHSPC) has rapidly evolved, with androgen deprivation therapy (ADT)-based combinations emerging as the new standard of care^3^. Several of these novel treatments target the androgen receptor-testosterone pathway, utilizing agents such as Androgen Receptor Pathway Inhibitors (ARPIs) that were initially developed for metastatic castration-resistant prostate cancer (mCRPC). Despite the rapid adoption of these intensified mHSPC drug combinations, progression to mCRPC is inevitable.

Currently, the prognostication in mCRPC relies primarily on clinical variables, either with CTC number (> 5 per 7.5 ml blood)^4^ or non-specific blood-based proteins including alkaline phosphatase (ALP), lactate dehydrogenase (LDH), albumin, and hemoglobin levels^5–7^. Clinical factors such as the Eastern Cooperative Oncology Group (ECOG) performance status, opioid use for pain management, and presence of visceral metastases are also included to derive prognostic risk groups in mCRPC^2^. While these markers offer the advantages of being validated in prospective randomized clinical trials and are easily measured in blood samples or clinical history, they are not mCRPC tumor biology-specific. Consequently, it is challenging to leverage prognostic risk groups developed from these markers to develop targeted therapeutic interventions in mCRPC patients with poor prognostic risk.

To address efforts to integrate blood molecular profiling with the non-specific blood-based biomarkers into prognostication have emerged in the last decade. Plasma cell-free DNA (cfDNA) alterations, circulating tumor DNA (ctDNA), and specific genomic aberrations such as *TP53* mutations, *AR* amplifications, and *RB1* loss have shown promise as prognostic classifiers in metastatic prostate cancer^8–13^. However, genetic alterations can be rare, arise later in tumor evolution, and exhibit heterogeneity across different tumor regions, which limits their sensitivity and specificity as biomarkers. DNA methylation has, also emerged as a promising biomarker due to its enhanced sensitivity and stability^14,15^. It is well known that DNA methylation changes arise early in tumorigenesis and span larger genomics regions that can be detected even at low tumor fractions^16^. Additionally, DNA methylation patterns are tissue-specific which provide insights into tissue of origin and improve cancer discrimination^17^.

In this study, we examined plasma cfDNA methylation profiles in 96 prostate cancer patients from the localized, organ-confined to metastatic states of prostate cancer progression. We identified methylation signatures specific to the state of prostate cancer progression and then developed epigenetic prognostic classifiers for mCRPC lethality by integrating clinical and epigenetic markers. Our goal was to establish a clinical-molecular tool specific to mCRPC-biology that enhance prognostication and potentially guides personalized targeted treatment strategies based on molecular pathways associated with the aggressive mCRPC state.

## Results

### Clinical characteristics of prostate cancer patients

We enrolled 96 prostate cancer patients after obtaining written informed consent with adequate longitudinal follow up for mCRPC patients. Details of the real-world database are provided under **Supplementary Methods**. Briefly, the patient cohort consisted of individuals with localized prostate cancer (n = 19), mHSPC (n = 28), and mCRPC (n = 49). Clinical variables were collected from Electronic Medical Records and cohort demographics are summarized in Table 1 including follow up periods for the mCRPC sub-cohort and the event rate of death for mCRPC patients. All 28 mHSPC patients provided blood samples prior to any androgen deprivation treatment. The mean yield of cfDNA was 18.96 nanograms of cfDNA per milliliter of plasma (ranging 2–612 ng/mL) (**Supplementary Data 1**).

### Mapping characteristics of targeted methylation sequencing

We performed Enzymatic Methyl-Seq (EM-Seq) to identify 5-methylcytosine (5-mC) at CpG sites in 437 target regions selected from peer-reviewed studies, TCGA data, and prostate cancer related oncogene and tumor-suppressor gene regions (**Supplementary Data 2**). Across our patient samples, we achieved means of 77.4% (ranging 48.4–80.3%) mappable reads, 60.5% (ranging 41–68%) on-target rate, and 1.9% (ranging 1.1–2.7%) duplicate alignments (**Supplementary Fig. 1**). Among the unique alignments in the target regions, we found that 99.3% had coverage greater than 10x and overall mean coverage was 96x (**Supplementary Fig. 2**).

### Distribution of methylation haplotype blocks

Following alignment and methylation calling with Bismark^18^, we utilized mHapSuite^19^ to conduct an exploration of methylation haplotype blocks (MHB), which we defined as genomic regions containing at least three bases and a linkage disequilibrium value (*r*^*2*^) greater than 0.3, across patients representing different states of prostate cancer. This analysis identified a total of 3,525 MHBs in the 437 target regions across all patient samples. After we excluded MHBs with less than 3 CpGs and median read counts below 50, 1,530 MHBs remained for further analysis. Among these, 130 MHBs were located within positive control regions, while 16 MHBs were within negative control regions (**Supplementary Fig. 3**). The median methylation was 84.6% among the positive control regions and 0.3% among the negative control regions (**Supplementary Fig. 4**).

Among the 437 target regions, 366 were involved in genes implicated in cancers, of which 263 were linked to prostate cancer based on prior peer-reviewed studies and known oncogenes and tumor suppressor genes (**Supplemental Data 2**). These 366 cancer-associated regions were represented by 1194 MHBs (**Supplementary Fig. 3**). The mean length of these MHBs was 127 base pairs (bp) (ranging from 5 to 2100 bp) and the mean number of CpGs per MHB was 11 CpGs (ranging from 3 to 137 CpGs) (**Supplementary Fig. 5**). Since MHBs are defined based on linkage disequilibrium and span contiguous genomic regions, they can overlap multiple functional elements and be categorized into more than one CpG or genic feature. The 1194 MHBs were primarily located in CpG islands (76.3% of MHBs) and CpG shores (30% of MHBs). Furthermore, these regions largely consisted of promoters (47.3% of MHBs), introns (68.6% of MHBs), exons (50.3% of MHBs), and genic regions 1 to 5 Kb from transcription start sites (TSS) (53.2% of MHBs) (Supplementary Fig. 6).

### Differential methylation haplotype analysis reveals progressive methylation changes throughout progression of prostate cancer

**Figure 1:**
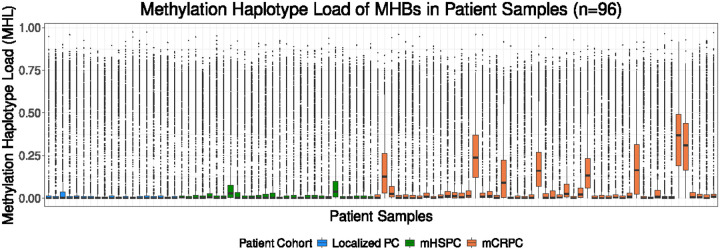
Methylation haplotype load increases with the advancement of prostate cancer. Box plot depicting the MHL for each MHB (n=1194) across the patients representing localized prostate cancer (blue, n=19), mHSPC (green, n=28), and mCRPC (orange, n=49). MHL methylation haplotype load, MHB methylation haplotype block, PC prostate cancer, mHSPC metastatic hormone sensitive prostate cancer, mCRPC metastatic castration resistant prostate cancer.

**Figure 2:**
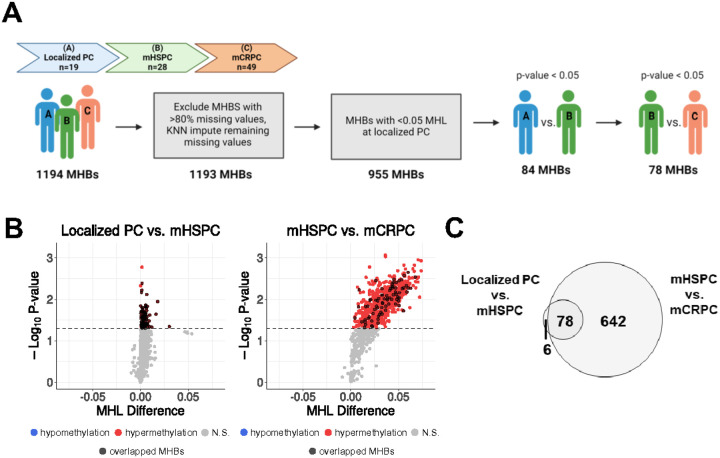
MHBs are differentially methylated across the states of prostate cancer.(A) Overview of the pipeline to identify MHBs and then perform comparative analyses between the progressive states of prostate cancer. (B) Volcano plots demonstrating the differentially methylated MHBs found when comparing localized PC to mHSPC and mHSPC to mCRPC. Hypomethylated MHBs are in blue, hypermethylated MHBs are in red, and MHBs shared across all states are found in black. (C) Venn diagram depicting the significant MHBs (p < 0.05) which are overlapping between the comparative analyses. MHL methylation haplotype load, MHBs methylation haplotype blocks, PC prostate cancer, mHSPC metastatic hormone sensitive prostate cancer, mCRPC metastatic castration resistant prostate cancer.

To investigate methylation changes across different prostate cancer progressive states, we calculated successive CpG methylation at varying fragment lengths by determining the methylation haplotype load (MHL) across the 1194 MHBs^19,20^. This analysis demonstrated increasing MHL values as the disease progressed to the metastatic prostate cancer states compared to localized organ-confined prostate cancer (Fig. 1). To identify differentially methylated regions (DMRs) among localized prostate cancer, mHSPC, and mCRPC states, we excluded a single MHB with greater than 80% missing MHL values and in the remaining samples imputed missing values with KNN imputation^21^. From the remaining 1193 MHBs, we further excluded 238 MHBs with MHL > 0.05 in localized prostate cancer. The rationale behind the filtering criteria is the low ctDNA fraction found in localized prostate cancer^22,23^ which could lead to low cancer-specific methylation detection in the localized state. High methylation level found at the localized state may represent contributions from non-tumor cells such as lymphocytes. Therefore, the remaining 955 MHBs with low successive methylation load (MHL ≤ 0.05) were used in subsequent analyses (Fig. 2A).

From the 955 MHBs, we identified 84 DMRs across localized prostate cancer and mHSPC (two-tailed Welch’s unpaired t-test, p-value < 0.05) (Fig. 2B). When comparing mHSPC with mCRPC, we identified 720 DMRs (two-tailed Welch’s unpaired t-test, p-value < 0.05), of which 78 DMRs were found in common with the analysis comparing localized prostate cancer with mHSPC (Fig. 2B, 2C). These DMRs all demonstrated a consistent positive shift in MHL values as the disease progressed, suggesting progressive hypermethylation within target regions.

### Target region methylation and clinical biomarkers are associated with overall survival

**Figure 3:**
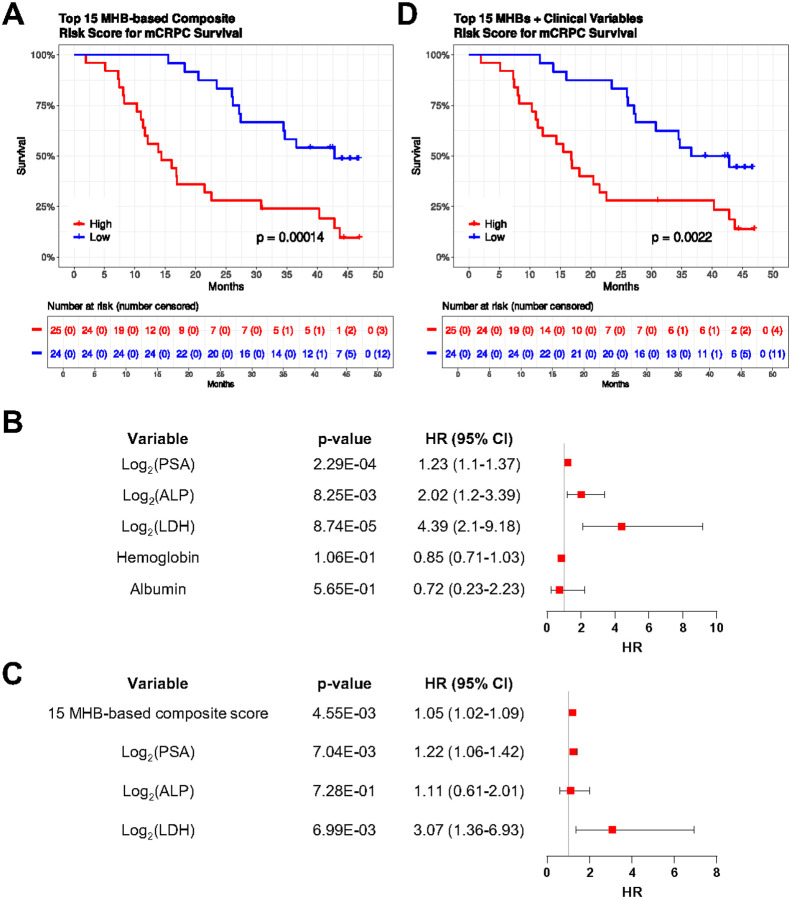
Top MHBs and clinical biomarkers are associated with mCRPC survival. (A) Kaplan-Meier plot showing mCRPC survival for patients stratified into high-risk and low-risk groups based on a 15 MHB composite score (log-rank test, p=0.00014). (B) Forest plot showing hazard ratios and 95% CI derived from univariate Cox analyses of clinical biomarkers. (C) Forest plot showing hazard ratios and 95% CI derived from multivariable Cox analysis. (D) Kaplan-Meier plot showing high-risk and low-risk patient groups based on a composite risk score derived from 15 MHBs and clinical biomarkers, and its association with mCRPC survival (log-rank test, p=0.0022). MHB methylation haplotype block, mCRPC metastatic castration resistant prostate cancer, PSA prostate specific antigen, ALP alkaline phosphatase, LDH lactate dehydrogenase, HR hazard ratio, CI confidence interval.

To investigate the prognostic potential of the identified DMRs with overall survival in mCRPC, we selected the top 20 of the 78 common DMRs identified in the comparative analysis between localized, mHSPC, and mCRPC states for further evaluation. Among the 49 mCRPC patients, 34 died during follow-up and had a median survival time of 17.5 months (Supplementary Data 1). Univariate Cox proportional hazards survival analyses showed that all selected 20 DMRs were significantly associated with overall survival (p-value < 0.05) (Table 2). After excluding DMRs associated with the same gene, the remaining 15 were used to generate an MHB-based composite prognostic risk score for mCRPC survival analysis. Patients with risk scores above the median risk score were classified as high-risk, while those with risk scores below the median were classified as having a low-risk prognostic score. We found that mCRPC patients with high-risk scores had significantly worse overall survival. Specifically, the median survival time of high-risk mCRPC patients was 14.4 months while the median survival time of low-risk patients was 42.8 months during the 47-month follow-up period (log-rank test, p-value = 0.00014) (Fig. 3A).

To assess the potential to enhance prognosis over current prognostic factors alone, the MHB-based composite score was integrated with known prognostic clinical biomarkers (PSA, LDH, ALP, albumin, and hemoglobin). In this dataset, on Univariate Cox analyses increased PSA, LDH, and ALP levels were associated with worse overall survival in mCRPC patients (p-value < 0.05) (Fig. 3B). In multivariable analysis on integrating the MHB-based composite score with the significant (univariate) clinical prognostic factors, we observed that the MHB-based score was the most significant variable (p-value = 0.00455) associated with mCRPC survival (Fig. 3C). At the multivariable model high-risk mCRPC patients had significantly worse overall survival than low-risk patients, with median survival times of 16.8 vs. 39.7 months respectively (log-rank test, p-value = 0.0022). (Fig. 3D).

### Nontumor-derived methylation regions also show prognostic potential

In addition to MHBs with low methylation, we also examined 238 MHBs with high successive methylation at localized prostate cancers (MHL > 0.05). Although these methylation signals were unlikely derived from tumor cells, the methylation changes in cfDNA could reflect tumor microenvironment and immune responses. Comparative analysis identified 39 DMRs between localized prostate cancer and mHSPC, of which 5 remained significant when comparing mHSPC with mCRPC (two-tailed Welch’s unpaired t-test, p-value < 0.05) (**Supplementary Fig. 7**). We further examined the prognostic potential of these MHBs and observed that 4 of the 5 MHBs were significantly associated with mCRPC survival in univariate Cox analysis (p-value < 0.05) (**Supplementary Data 3**). The MHB-based composite risk score based on the top 3 DMRs also significantly distinguished mCRPC patients with different survival risks (**Supplementary Fig. 8**).

### Methylation markers correlate with ctDNA fraction

**Figure 4:**
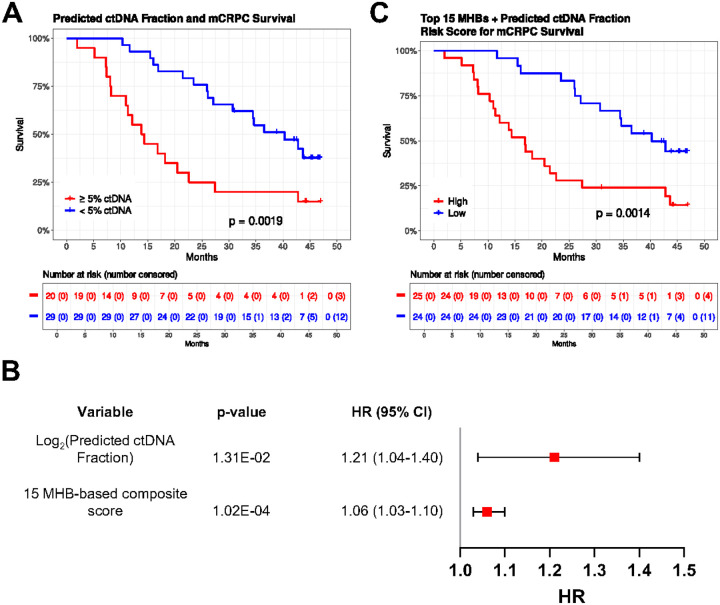
ctDNA fraction and top MHBs are associated with mCRPC survival. (A) Kaplan-Meier showing mCRPC survival probability based on high and low ctDNA fraction (log-rank test, p=0.0019). (B) Kaplan-Meier plot showing high-risk and low-risk patient groups based on a composite risk score derived from predicted ctDNA fraction and 15 TD MHBs, and its association with mCRPC survival (log-rank test, p=0.0014). (C) Forest plot showing hazard ratios and 95% CI derived from multivariable Cox analysis. MHB methylation haplotype block, mCRPC metastatic castration resistant prostate cancer, HR hazard ratio, CI confidence interval.

To explore whether ctDNA fraction might influence survival predictions in this patient cohort, we utilized the online ctDNA fraction prediction tool in mCRPC state (https://www.ctdna.org)^24^. Predicted ctDNA fraction of patient samples was performed using the concentrations of plasma cfDNA, PSA, LDH, and ALP (**Supplementary Data 1**). Considering that ctDNA fraction can reflect tumor burden and disease progression^25–27^, we assessed whether its variation among patients would be reflected in survival predictions. In line with previous studies in mCRPC^12,28,29^ demonstrating that high ctDNA fraction was observed to be associated with poor prognosis our study also showed that the estimated ctDNA fraction was highly associated with mCRPC survival. Patients with low ctDNA fraction (< 5%) had a median survival time of 42.8 months and patients with high ctDNA fraction (≥ 5%) had a median survival time of 16.8 months (log-rank test, p-value = 0.0019) (Fig. 4A). We further evaluated whether MHL values in the MHBs correlated with the predicted ctDNA fraction. We found that the MHL values in 72.7% MHBs correlate with ctDNA fraction (Pearson r > 0.5) among the patients with the ctDNA fraction above 1% (n = 32). (**Supplementary Data 4, Supplementary Fig. 10**). When examining the top 20 most correlated MHBs, we observed their significant association with mCRPC survival (**Supplementary Data 5**).

### cfDNA methylation is an independent predictor for survival in a multimodal risk model

Clinical variables, ctDNA fraction and cfDNA methylation observed to be associated with survival were integrated into a single multi-modal model for prognostic performance. We first combined the ctDNA fraction and the 15 MHB-based composite score into a multivariable composite risk score. Multivariable analysis demonstrated that both factors were independently associated with overall survival (Fig. 4B). The combined model efficiently stratified low-risk and high-risk patients, with a median survival time of 40.3 months in low-risk patients and 16.8 months in high-risk patients (log-rank test, p-value = 0.0014) (Fig. 4C). We then incorporated the clinical prognostic biomarkers of PSA, LDH, and ALP into the multivariable composite score to establish a multi-modal risk score. Among all survival-associated variables, the 15 MHB-based composite score remained significantly predictive of mCRPC survival (p-value = 0.00434) (**Supplementary Fig. 9A**). By integrating ctDNA fraction, methylation, and clinical biomarkers, the multi-modal risk score classified survival with the median survival time for the low-risk and the high-risk groups observed to be 42.8 months and 15.5 months, respectively (log-rank test, p-value = 0.00025) (**Supplementary Fig. 9B**). These findings suggest that methylation markers correlated with ctDNA fraction hold promise as complementary biomarkers for survival prediction in mCRPC, especially in cases where ctDNA fractions are below the limit of detection.

### Prognostic multi-modal nomogram for mCRPC survival

**Figure 5:**
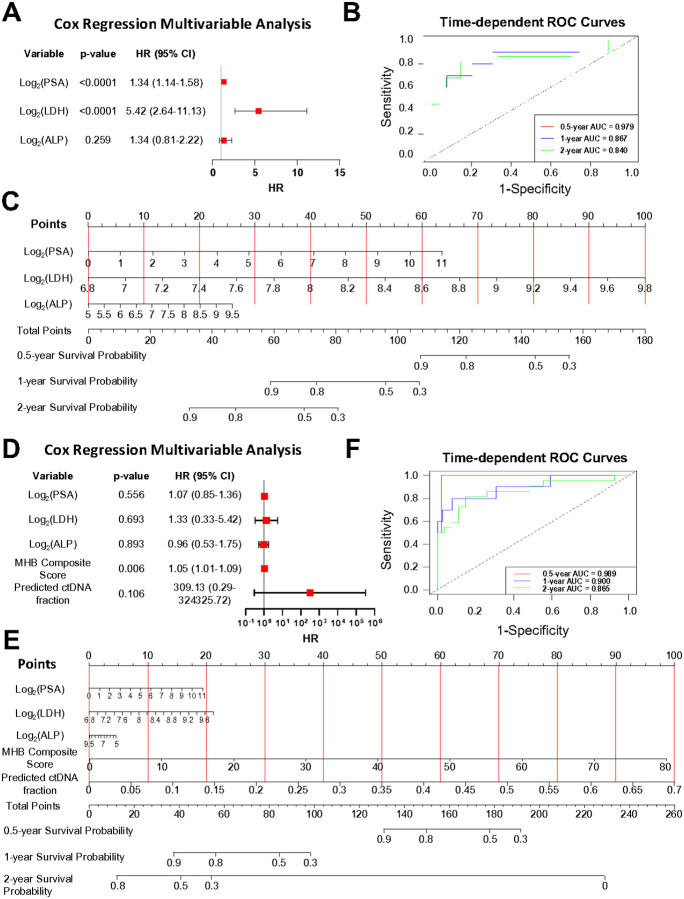
Nomograms built from clinical variables and MHBs predict probability of survival. (A) Forest plot summarizing the HR for PSA, LDH, and ALP. (B) Time-dependent ROC AUC for 6 months survival, 12 months survival, and 24 months survival based on multivariable model utilizing PSA, LDH, and ALP. (C) Nomogram computing for 0.5-year, 1-year, and 2-year probability based on PSA, LDH, and ALP levels. (D) Forest plot summarizing the HR for PSA, LDH, ALP, MHB-based composite score, and predicted ctDNA fraction. (E) Nomogram computing for 0.5-year, 1-year, and 2-year probability based on clinical variables, MHB-based composite score, and predicted ctDNA fraction. (F) ROC AUC for 6 months survival, 12 months survival, and 24 months survival based on multivariable model utilizing clinical variables, MHB-based composite score, and predicted ctDNA fraction. HR hazard ratio, ROC AUC Receiver Operating Characteristic Area Under the Curve, PSA prostate specific antigen, ALP alkaline phosphatase, LDH lactate dehydrogenase, HR hazard ratio, CI confidence interval.

Instead of risk-group categorizations, we identified individual mCRPC patient survival outcome for patient centricity based on the multi-modal model. Clinical biomarkers (PSA, LDH, and ALP) associated with mCRPC survival at the univariate level (p-value < 0.05) (Fig. 3B) were included in a multivariable analysis. Only PSA and LDH were observed to remain significantly associated with survival (p-value < 0.0001) (Fig. 5A) and were included in a prognostic nomogram. The performance of the clinical biomarker-based nomogram model was assessed with time-dependent receiver operating characteristic (ROC) analysis with area under the curve (AUC) values of 0.98 for 6-month survival, 0.87 for 1-year survival, and 0.84 for 2-year survival (Fig. 5B). The corresponding nomogram utilizing clinical biomarkers alone to predict survival at these time points is presented in Fig. 5C.

Combining the 15 MHB-based composite score and the ctDNA fraction with the clinical biomarkers after multivariable analysis the 15 MHB-based composite score was the only significantly associated variable with survival (p-value = 0.006). The predicted ctDNA fraction was not significant (p-value = 0.106) but had an increased hazard ratio (HR) (309.13) with a wide 95% confidence interval (0.29–324,325.72) (Fig. 5D). Th integrated clinical biomarker, 15 MHB-based composite score and ctDNA fraction nomogram for predicting 6-month, 1-year, and 2-year survival (Fig. 5E) showed a marginally higher predictive performance compared to the nomogram utilizing clinical biomarkers alone with AUC values increased to 0.99 for 6-month survival, 0.90 for 1-year survival, and 0.87 for 2-year survival (Fig. 5F).

## Discussion

In this study, we profiled tumor-specific cfDNA methylation patterns to identify the DMRs that provide insights into prostate cancer progression, survival prognosis, and risk stratification. Our findings reveal that a set of MHBs undergo epigenetic changes in advanced prostate cancer states. While it is not clear if the distinct patterns of hypermethylation in MHBs reflect prostate cancer biology, the MHB-based composite risk score did show a significant association with mCRPC survival. We also observed that cfDNA methylation in a significant number of target regions was correlated positively with predicted ctDNA fraction, which is associated with mCRPC survival. Finally, we integrated current prognostic clinical variables into the MHB-based risk score to develop patient-centric prognostic nomograms for individual mCRPC patients.

Methylation changes occur in larger genomic regions and often affect multiple CpG sites within a locus, generating a stronger cumulative signal that enhances detection sensitivity^30^. Importantly, DNA methylation changes occur early in tumorigenesis^31,32^ and can change to reflect environmental and treatment exposures^33,34^, while still expressing tissue-specific or tumor-specific patterns that allow for identification of the tissue of origin^17,35^. This makes methylation profiling an attractive biomarker strategy particularly when ctDNA fractions are low and fall below the detection limits of conventional ctDNA quantification methods, leading to false negatives. These quantification methods have been well reported using copy number variations (CNVs)^36–38^ and specific mutations^39–41^ but have limitations as they may lack sensitivity (CNV-based) or require high sequencing depth to capture rare mutant alleles^42^ because of variability of mutations across tumor types and disease states^43^. The identification of methylation markers potentially has a more sensitive readout of tumor burden and offer insights into dynamic tumor biology, which could inform treatment selection based on the underlying epigenetic landscape.

To facilitate clinical translation, this study developed nomograms as patient-centric tools to predict mCRPC survival. Prognostic risk groups can classify patients into broad groups based on categorical variables by simplifying continuous data into categories. While this is helpful, it does not account for the unique combination of clinical variables present in each patient and potentially overlooks patient-level nuances. Unlike categorical risk-grouping, a nomogram-based approach handles categorical and continuous variables more efficiently. Nomograms offer a visual, individualized risk prediction approach that integrates multiple prognostic variables, making them more interpretable and clinically actionable than standard risk models^44,45^. Although nomograms can be built using clinical variables alone, our study highlighted that the inclusion of tumor-specific methylation markers obtained through non-invasive blood draws enhances the model’s prognostic accuracy.

The potential of cfDNA methylation as a critical biomarker in assessing treatment response and clinical outcomes cfDNA methylation in prostate cancer detection, prognosis, and monitoring^46–49^ has been reported. In one study dynamic methylated changes associated with disease progression and treatment response in metastatic prostate cancer^46^ were observed. An independent study demonstrated that methylation patterns can serve as a proxy for tumor fraction in metastatic prostate cancer^47^. A tumor tissue-informed approach showed that limited methylation markers are capable of differentiating treatment-responsive from non-responsive mCRPC patients and predicting progression-free survival in mCRPC patients^48^. Lastly, a study employing a genome-wide approach identified methylation signatures that could distinguish between localized and metastatic cancer. Furthermore, methylation level at a gene promoter region was associated with progression-free survival and overall survival^49^. By incorporating cfDNA methylation into an integrative prognostic model, our current study explored a patient-centric application for cfDNA methylation in mCRPC prognostication.

There are several advantages to using a targeted rather than a whole genome methylation sequencing approach. First, the targeted EM-seq is more sensitive, cost-effective, and avoids DNA degradation associated with bisulfite-based sequencing approaches^50,51^. This enhanced sensitivity allows for the detection of subtle methylation differences that may be missed by traditional methods. Second, prior studies have typically analyzed mean methylation levels across sequencing reads within predefined genomic windows or specific target regions^47,48,52^. This approach may overlook the heterogeneity of methylation patterns within individual DNA fragments, further masking variations in methylation haplotypes that may have functional relevance in cancer progression. In our study, we leveraged MHBs which captured the coordinated methylation patterns across multiple CpGs within the same DNA fragment^20^ and in different states of cancer progression. By considering read-level methylation haplotypes and including samples collected from different progressive cancer states, a more granular view of regions undergoing tumor-specific epigenetic alterations in mCRPC could be obtained since the MHBs also considers interactions between CpG sites. Third, we generated a patient-centric prognostic nomogram, a methodology which has not commonly employed in prior cfDNA methylation studies, providing an intuitive, clinically actionable framework for survival prediction.

Despite the promising findings, our study has several limitations. The small sample size may limit the generalizability of our results, and future validation in larger, independent cohorts is necessary to confirm the study approach and the robustness of the prognostic models. Additionally, our study relied on predicted ctDNA fraction based on the measurement of clinical biomarkers rather than direct measurement, which may introduce uncertainty regarding the relationship between methylation changes and ctDNA levels. A direct ctDNA quantification alongside methylation profiling could provide a better understanding of their relationship. While our study has a small sample size, the study approach highlights potential cfDNA methylation profiling signals as clinically relevant tools for prognostication and disease monitoring in prostate cancer. These cfDNA methylation signatures could complement existing prognostic models to offer tumor-biology-based prognostic tools. Since cfDNA-based testing is non-invasive, scalable, and allows for longitudinal monitoring without the need for tissue biopsies, if validated this approach will aid in the development of patient-centric, biology-informed nomograms for individualized risk stratification.

## Methods

### Patient cohort

This study used plasma samples from a prospective, clinically annotated, real-world patient biobank. To avoid preanalytical variations, the sample collection followed uniform standard operating procedures (SOPs). These SOPs for blood collections, IRB approvals, cfDNA extraction methods are detailed under “Supplementary Methods”.

### Capture probe design

437 targeted methylation regions were selected from three major sources, including 1) Literature search identified 184 Differentially Methylated regions (DMRs) associated with prostate cancer; 2) TCGA-PRAD data showed 103 DMRs when compared to normal controls; and 3) Prostate cancer-related oncogenes and tumor suppressor genes (n = 79) (**Supplementary Data 2**). We also included 29 positive and 42 negative control regions. The positive and negative control regions were selected based on regions that consistently show > 95% methylation level and < 0.5% methylation level in cfDNA from healthy controls^17^, respectively. Overall, we selected 437 targeted regions, including 366 prostate cancer-related regions, 29 positive control, and 42 negative control regions. The selected regions covered all CpG islands of targeted genes and +/−5Kb flanking sequences. All capture probes were designed by Twist Custom Design Service.

### EM-seq library preparation and target capture

10–20 ng of cfDNA was subjected to Enzymatic Methyl-seq (EM-seq) (NEB, #E7120S). Briefly, the cfDNA was first ligated with methylated adaptors, followed by oxidation using TET2 reaction and deamination using APOBEC reaction. Indexed primers were then used for 8–10 cycles of amplification. To enrich regions of interest, whole genome sequencing libraries were captured with a custom-made Twist targeted methylome panel covering 3.44 Mbp of genome. The captured libraries were further amplified for an additional 5 cycles before performing 150bp paired-end (PE) sequencing in an Illumina sequencer.

### Data preprocessing and methylation calling

Quality control checks were performed with fastqc (v0.11.9) and multiqc (v1.12) on the FASTQ files. Illumina adapter sequences were trimmed with cutadapt (v2.8), with a filter applied to discard reads shorter than 20 bp. Alignment to the human reference genome hg38/GRCh38 and methylation calling were performed on the processed reads using Bismark (v0.22.3) with bowtie2 (v2.3.5.1)^18^. Alignment parameters were set to a multi-seed length of 22 bp with 1 mismatch (-L 11, -N 1). Deduplication to remove alignments at the same position in the mapped genome was performed with Bismark (deduplicate_bismark). Mappable reads and duplicate alignments were extracted from Bismark alignment reports. To evaluate sequencing coverage in target regions, mosdepth (v0.3.3) was utilized to generate coverage profiles for each sample.

### Methylation haplotype blocks (MHBs) identification and methylation haplotype load (MHL) analysis

Methylation haplotypes were extracted by converting deduplicated BAM files into mHAP format with mHapSuite (v2.1) (convert), available at https://github.com/yoyoong/mHapSuite^19^. MHBs were then identified with mHapSuite (MHBDiscovery) within the target regions (± 5Kb) across all patients, based on the parameters of a core window of 3, and r^2^ cutoff of 0.3, and p-value cutoff of 0.05. MHL values were determined by calculating the methylation statistics of MHBs using mHapSuite (stat). MHL determines the level of consecutive CpG methylation at different lengths. MHL is calculated as

$$\text{MHL}=\frac{\sum_{i=1}^{10}i\times P(MH_{i})}{\sum_{i=1}^{10}i}$$

Where *P* (*MH*_*i*_) is the fraction of consecutively methylated CpGs at length *i*. For haplotypes with more than 10 consecutive CpGs, all lengths from 1 to 10 are considered. Identified MHBs were further filtered to retain only those with greater than 3 CpG sites and greater than 50 median reads across all patients. MHBs that were overlapping with positive and negative control regions were excluded from subsequent analyses for determining differential MHBs.

### Identification of differential MHBs

Prior to identifying which MHBs are differentially methylated, MHBs with greater than 80% missing MHL values were excluded. K-nearest neighbors (KNN) imputation using the caret (v6.0.94) R package was applied for the remaining MHBs with missing values. Welch’s t-tests were performed for the comparison of MHBs between patient cohorts (localized PC vs. mHSPC, and mHSPC vs. mCRPC). MHBs where the mean MHL values were less than 0.05 in localized PC patients were used for subsequent comparative analyses. MHBs with a p-value < 0.05 were deemed statistically significant. Volcano plots displaying the mean differences between localized PC vs. mHSPC and mHSPC vs. mCRPC were created using the tidyverse (v2.0.0) R package. The Venn diagrams detailing the overlaps between the two comparisons were created using the VennDiagram (v1.7.3) R package.

### Survival analyses

Survival probabilities for each MHB were assessed using multiple univariate Cox proportional hazards models with the survival (v3.5.8) R package. A reduced panel of MHBs was selected based on the top statistically differentially methylated MHBs, which overlapped in all t-test comparisons and had Cox model p-value < 0.05. For MHBs representing the same gene region, the MHB with the highest hazard ratio was selected for the panel. A composite score (*C*) for a panel of MHBs was calculated as the sum of the products the Cox coefficient (*β*) and the corresponding MHL value (*M*) for each MHB (*C* = *β*_1_*M*_1_ +*β*_2_*M*_2_ …). Risk scores (*R*) incorporating a panel of MHBs represented by their composite score and select clinical biomarkers (*V*) were calculated by multiplying the multivariable Cox coefficient for each covariate with its associated value and summing the resulting products (*R* = *β*_1_*C*_1_ +*β*_1_*V*_1_ + *β*_2_*V*_2_ …). For a given model, risk scores which were greater than the median risk score were categorized as high-risk while risk scores lower than the median risk score were categorized as low-risk. Clinical biomarkers were log-transformed by a base of 2 for survival analyses. Differences in survival probability were evaluated with Wald test, with a p-value < 0.05 considered to be statistically significant. Kaplan-Meier plots to represent the differences in survival probability were generated using the survminer (v0.4.9) R package.

### Prediction of ctDNA fraction and correlation with MHL

Predicted plasma ctDNA fractions for mCRPC patients (n = 49) were determined by inputting cfDNA, PSA, LDH, and ALP information for each patient into the online tool https://www.ctdna.org^24^. Patients with 0–1% predicted ctDNA fraction (n = 17) were excluded from correlation analyses. Among the remaining mCRPC patients (n = 32) Pearson correlation was performed between the predicted ctDNA fraction and MHL of each MHB that had greater than 50% non-missing values. A p-value < 0.05 and Pearson r > 0.5 were applied as cut-offs to determine the MHBs that were highly correlated with predicted ctDNA fraction. The plots depicting the correlation between ctDNA fraction and highly correlated MHBs and generated with the tidyverse and ggpubr (v0.6.0) R package.

### Nomogram Development

To develop the nomogram, Univariate Cox regression analyses were first used to identify potential predictors into multivariate Cox regression, which estimated HR for each predictor. Then, the multivariate Cox regression analysis and stepwise selection were utilized to identify the independent predictors for predicting overall survival (OS). Finally, these identified predictors were applied to build a Cox regression model. A survival nomogram was built based on a Cox regression model to estimate OS probabilities, examining the relationship between survival and multiple predictors. The nomogram used the model’s coefficients to assign points to each predictor and estimate survival probabilities for individuals. Model performance was evaluated using the AUC.

The nomograms were built based on the identified independent predictors for predicting the 6-, 12- and 24-month OS, and each predictor was represented on a separate axis in the nomogram. According to the nomograms constructed, the 6-, 12- and 24-month probability of OS could be acquired by calculating the total score of all independent variables. First, for each predictor, draw a vertical line to the ‘points’ scale at the top of the nomogram, then, sum the points from each predictor to get a total point score, next, on the ‘total points’ scale, draw a vertical line from the total points score scale to the survival probability scales, where the intersection indicates the patient’s predicted probability of survival. All analyses were conducted in RStudio. The nomogram was constructed using the ‘nomogram’ function from the ‘rms’ package. Statistical significance was defined by a two-sided p-value < 0.05.

## Figures and Tables

**Table 1:** Clinical characteristics of patients in different states of cancer progression

	Localized PC	mHSPC	mCRPC
Total Patients	19	28	49
Age at sample collection (years)	68 (57–85)	73.5 (58–85)	70 (53–86)
PSA at sample collection (ng/mL)	2.9 (0–38.1)	9.15 (0.01–1401.8)	7.5 (0.1–1863.6)
ALP at sample collection (U/L)	69 (44–492)	77 (47–987)	88 (41–639)
LDH at sample collection (U/L)	198 (150–276)	202 (131–442)	217 (122–811)
Hemoglobin at sample collection	14.6 (7.8–18)	15.05 (12.4–18.1)	13.1 (8.6–16)
Albumin at time of sample collection	4.1 (3.4–4.8)	4.05 (3.6–4.6)	4 (3.5–4.7)
Metastatic Volume at mHSPC			
Low	0	13	12
High	0	2	11
Unknown	19	13	26
Gleason Score at ID			
≤7	9	11	18
≥8	10	16	31
Clinical T Staging at initial presentation			
T1	4	2	2
T2	7	9	14
T3	8	7	8
T4	0	3	3
TX	0	7	19
T-unknown	0	0	3
Median mHSPC follow-up time from sample collection to last follow-up (months)	NA	42.25 (13.34–46.92)	NA
Median time from first mHSPC treatment to failure (months)	NA	10.53 (3.2–42.23)	13.07 (3.1–82.03)
mHSPC Patients progressed on ADT by date of analysis	NA	7	NA
Median mCRPC follow-up time from sample collection to date of analysis (months)	NA	NA	27.11 (1.94–46.92)
Median follow-up time from date of mCRPC to last follow-up (months)	NA	NA	50.2 (11.3–199.37)
Patients deceased by date of analysis	1	5	34

a Data are median (range); *PC* prostate cancer, *mHSPC* metastatic hormone sensitive prostatecancer, *mCRPC* metastatic castration resistant prostate cancer, *PSA* prostate specific antigen, *ALP* alkaline phosphatase, *LDH* lactate dehydrogenase, *ID* initial diagnosis.

**Table 2:** Significant association of top 20 MHBs with mCRPC survival.

Gene	MHB Region	p-value	HR (95% CI)
ALOX5	chr10:45418808–45420022	2.34E-07	2.27 (1.66–3.09)
HIC1	chr17:2056975–2057271	1.96E-07	2.45 (1.75–3.43)
HIC1	chr17:2058035–2058073	1.10E-07	2.66 (1.85–3.82)
TMEM106A	chr17:43211484–43212450	2.52E-08	2.82 (1.96–4.06)
HOXB5	chr17:48596419–48596858	1.74E-07	2.48 (1.76–3.49)
cg15222899	chr18:57427748–57428143	1.51E-07	2.32 (1.69–3.18)
WFDC2	chr20:45469884–45470211	1.43E-07	2.47 (1.76–3.45)
EFEMP1	chr2:55923171–55923364	1.91E-06	2.27 (1.62–3.18)
SOX11	chr2:5691072–5691083	1.23E-07	3.18 (2.07–4.89)
cg09064304	chr4:140498280–140498336	2.07E-08	3.29 (2.17–4.98)
cg09064304	chr4:140498337–140498442	1.40E-08	2.97 (2.04–4.33)
SCGB3A1	chr5:180591347–180591432	7.47E-08	2.63 (1.85–3.75)
SCGB3A1	chr5:180591433–180591585	5.90E-08	2.57 (1.83–3.62)
SCGB3A1	chr5:180591586–180591782	1.12E-07	2.58 (1.82–3.66)
FAM115A	chr7:143885367–143885557	2.80E-07	2.63 (1.82–3.8)
KCNH2	chr7:150958529–150958549	8.07E-08	3 (2.01–4.49)
PTPRN2	chr7:157686104–157686172	7.04E-08	2.44 (1.76–3.38)
PTPRN2	chr7:157691808–157692130	6.90E-08	2.45 (1.77–3.39)
PRDM14	chr8:70070419–70070468	6.16E-08	2.46 (1.78–3.41)
CDKN2A	chr9:21989731–21989796	4.43E-05	1.87 (1.39–2.53)

*MHBs* **methylation haplotype blocks**, *HR* **Hazard Ratio**, *CI* **Confidence Interval**, *mCRPC* **metastatic castration resistant prostate cancer**.

## Data Availability

The human reference genome hg38 was downloaded from UCSC. De-identified sequencing data from patients in this study is available on request.

## References

[1] Bray F. Global cancer statistics 2022: GLOBOCAN estimates of incidence and mortality worldwide for 36 cancers in 185 countries. CA Cancer J Clin 74, 229–263 (2024). 10.3322/caac.21834 38572751

[2] Halabi S. External Validation of a Prognostic Model of Overall Survival in Men With Chemotherapy-Naive Metastatic Castration-Resistant Prostate Cancer. J Clin Oncol 41, 2736–2746 (2023). 10.1200/JCO.22.02661 37040594 PMC10414709

[3] Omrcen T. Systemic Triple Therapy in Metastatic Hormone Sensitive Prostate Cancer (Mhspc). Acta Clin Croat 61, 81–85 (2022). 10.20471/acc.2022.61.s3.12 36938560 PMC10022414

[4] Gorin M. A. Circulating tumour cells as biomarkers of prostate, bladder, and kidney cancer. Nat Rev Urol (2016). 10.1038/nrurol.2016.224 27872478

[5] Halabi S. Overall Survival of Black and White Men With Metastatic Castration-Resistant Prostate Cancer Treated With Docetaxel. J Clin Oncol 37, 403–410 (2019). 10.1200/JCO.18.01279 30576268 PMC6804881

[6] Halabi S. Updated prognostic model for predicting overall survival in first-line chemotherapy for patients with metastatic castration-resistant prostate cancer. J Clin Oncol 32, 671–677 (2014). 10.1200/JCO.2013.52.3696 24449231 PMC3927736

[7] Halabi S. Prognostic model predicting metastatic castration-resistant prostate cancer survival in men treated with second-line chemotherapy. J Natl Cancer Inst 105, 1729–1737 (2013). 10.1093/jnci/djt280 24136890 PMC3833929

[8] Huang J. Plasma Copy Number Alteration-Based Prognostic and Predictive Multi-Gene Risk Score in Metastatic Castration-Resistant Prostate Cancer. Cancers (Basel) 14 (2022). 10.3390/cancers14194714 PMC9562906 36230636

[9] Kwan E. M. Plasma Cell-Free DNA Profiling of PTEN-PI3K-AKT Pathway Aberrations in Metastatic Castration-Resistant Prostate Cancer. JCO Precis Oncol 5 (2021). 10.1200/PO.20.00424 PMC8232889 34250422

[10] Huang X. Exosomal miR-1290 and miR-375 as prognostic markers in castration-resistant prostate cancer. Eur Urol 67, 33–41 (2015). 10.1016/j.eururo.2014.07.035 25129854 PMC4252606

[11] Kohli M. Prognostic association of plasma cell-free DNA-based androgen receptor amplification and circulating tumor cells in pre-chemotherapy metastatic castration-resistant prostate cancer patients. Prostate Cancer Prostatic Dis 21, 411–418 (2018). 10.1038/s41391-018-0043-z 29858592 PMC6126974

[12] Kohli M. Clinical and genomic insights into circulating tumor DNA-based alterations across the spectrum of metastatic hormone-sensitive and castrate-resistant prostate cancer. EBioMedicine 54, 102728 (2020). 10.1016/j.ebiom.2020.102728 32268276 PMC7186589

[13] Romanel A. Plasma AR and abiraterone-resistant prostate cancer. Sci Transl Med 7, 312re310 (2015). 10.1126/scitranslmed.aac9511 PMC6112410 26537258

[14] Wong J., Muralidhar R., Wang L. & Huang C. C. Epigenetic modifications of cfDNA in liquid biopsy for the cancer care continuum. Biomed J 48, 100718 (2024). 10.1016/j.bj.2024.100718 38522508 PMC11745953

[15] Strand S. H., Orntoft T. F. & Sorensen K. D. Prognostic DNA methylation markers for prostate cancer. Int J Mol Sci 15, 16544–16576 (2014). 10.3390/ijms150916544 25238417 PMC4200823

[16] Luo H., Wei W., Ye Z., Zheng J. & Xu R. H. Liquid Biopsy of Methylation Biomarkers in Cell-Free DNA. Trends Mol Med 27, 482–500 (2021). 10.1016/j.molmed.2020.12.011 33500194

[17] Moss J. Comprehensive human cell-type methylation atlas reveals origins of circulating cell-free DNA in health and disease. Nat Commun 9, 5068 (2018). 10.1038/s41467-018-07466-6 30498206 PMC6265251

[18] Krueger F. & Andrews S. R. Bismark: a flexible aligner and methylation caller for Bisulfite-Seq applications. Bioinformatics 27, 1571–1572 (2011). 10.1093/bioinformatics/btr167 21493656 PMC3102221

[19] Hong Y. mHapBrowser: a comprehensive database for visualization and analysis of DNA methylation haplotypes. Nucleic Acids Res 52, D929–D937 (2024). 10.1093/nar/gkad881 37831137 PMC10767976

[20] Guo S. Identification of methylation haplotype blocks aids in deconvolution of heterogeneous tissue samples and tumor tissue-of-origin mapping from plasma DNA. Nat Genet 49, 635–642 (2017). 10.1038/ng.3805 28263317 PMC5374016

[21] Kuhn M. Building Predictive Models in R Using the caret Package. J Stat Softw 28, 1–26 (2008). https://doi.org/DOI 10.18637/jss.v028.i0527774042

[22] Hennigan S. T. Low Abundance of Circulating Tumor DNA in Localized Prostate Cancer. JCO Precis Oncol 3 (2019). 10.1200/PO.19.00176 PMC6746181 31528835

[23] Lau E. Detection of ctDNA in plasma of patients with clinically localised prostate cancer is associated with rapid disease progression. Genome Med 12, 72 (2020). 10.1186/s13073-020-00770-1 32807235 PMC7430029

[24] Fonseca N. M. Prediction of plasma ctDNA fraction and prognostic implications of liquid biopsy in advanced prostate cancer. Nat Commun 15, 1828 (2024). 10.1038/s41467-024-45475-w 38418825 PMC10902374

[25] Lipson E. J. Circulating tumor DNA analysis as a real-time method for monitoring tumor burden in melanoma patients undergoing treatment with immune checkpoint blockade. J Immunother Cancer 2, 42 (2014). 10.1186/s40425-014-0042-0 25516806 PMC4267741

[26] Smith J. T. Circulating Tumor DNA as a Biomarker of Radiographic Tumor Burden in SCLC. JTO Clin Res Rep 2, 100110 (2021). 10.1016/j.jtocrr.2020.100110 34589992 PMC8474385

[27] Kirchweger P. Circulating tumor DNA correlates with tumor burden and predicts outcome in pancreatic cancer irrespective of tumor stage. Eur J Surg Oncol 48, 1046–1053 (2022). 10.1016/j.ejso.2021.11.138 34876329

[28] Annala M. Evolution of Castration-Resistant Prostate Cancer in ctDNA during Sequential Androgen Receptor Pathway Inhibition. Clin Cancer Res 27, 4610–4623 (2021). 10.1158/1078-0432.CCR-21-1625 34083234

[29] Nørgaard M. Prognostic Value of Low-Pass Whole Genome Sequencing of Circulating Tumor DNA in Metastatic Castration-Resistant Prostate Cancer. Clin Chem 69, 386–398 (2023). 10.1093/clinchem/hvac224 36762756

[30] Keller L., Belloum Y., Wikman H. & Pantel K. Clinical relevance of blood-based ctDNA analysis: mutation detection and beyond. Br J Cancer 124, 345–358 (2021). 10.1038/s41416-020-01047-5 32968207 PMC7852556

[31] Baylin S. B. Aberrant patterns of DNA methylation, chromatin formation and gene expression in cancer. Hum Mol Genet 10, 687–692 (2001). 10.1093/hmg/10.7.687 11257100

[32] Kanwal R., Gupta K. & Gupta S. Cancer epigenetics: an introduction. Methods Mol Biol 1238, 3–25 (2015). 10.1007/978-1-4939-1804-1_1 25421652

[33] Sutton L. P. DNA methylation changes following DNA damage in prostate cancer cells. Epigenetics 14, 989–1002 (2019). 10.1080/15592294.2019.1629231 31208284 PMC6691980

[34] Pedersen C. A. DNA methylation changes in response to neoadjuvant chemotherapy are associated with breast cancer survival. Breast Cancer Res 24, 43 (2022). 10.1186/s13058-022-01537-9 35751095 PMC9233373

[35] Loyfer N. A DNA methylation atlas of normal human cell types. Nature 613, 355–364 (2023). 10.1038/s41586-022-05580-6 36599988 PMC9811898

[36] Carter S. L. Absolute quantification of somatic DNA alterations in human cancer. Nat Biotechnol 30, 413–421 (2012). 10.1038/nbt.2203 22544022 PMC4383288

[37] Ha G. TITAN: inference of copy number architectures in clonal cell populations from tumor whole-genome sequence data. Genome Res 24, 1881–1893 (2014). 10.1101/gr.180281.114 25060187 PMC4216928

[38] Adalsteinsson V. A. Scalable whole-exome sequencing of cell-free DNA reveals high concordance with metastatic tumors. Nat Commun 8, 1324 (2017). 10.1038/s41467-017-00965-y 29109393 PMC5673918

[39] Newman A. M. An ultrasensitive method for quantitating circulating tumor DNA with broad patient coverage. Nat Med 20, 548–554 (2014). 10.1038/nm.3519 24705333 PMC4016134

[40] Cohen J. D. Detection and localization of surgically resectable cancers with a multi-analyte blood test. Science 359, 926–930 (2018). 10.1126/science.aar3247 29348365 PMC6080308

[41] Husain H. Tumor Fraction Correlates With Detection of Actionable Variants Across > 23,000 Circulating Tumor DNA Samples. JCO Precis Oncol 6, e2200261 (2022). 10.1200/PO.22.00261 36265119 PMC9616642

[42] Taylor A. M. Genomic and Functional Approaches to Understanding Cancer Aneuploidy. Cancer Cell 33, 676–689 e673 (2018). 10.1016/j.ccell.2018.03.007 29622463 PMC6028190

[43] Rolfo C. D. Measurement of ctDNA Tumor Fraction Identifies Informative Negative Liquid Biopsy Results and Informs Value of Tissue Confirmation. Clin Cancer Res 30, 2452–2460 (2024). 10.1158/1078-0432.CCR-23-3321 38526394 PMC11145175

[44] Balachandran V. P., Gonen M., Smith J. J. & DeMatteo R. P. Nomograms in oncology: more than meets the eye. Lancet Oncol 16, e173–180 (2015). 10.1016/S1470-2045(14)71116-7 25846097 PMC4465353

[45] Zheng H. Nomograms for prognostic risk assessment in glioblastoma multiforme: Applications and limitations. Clin Genet 102, 359–368 (2022). 10.1111/cge.14200 35882630

[46] Silva R. Longitudinal analysis of individual cfDNA methylome patterns in metastatic prostate cancer. Clin Epigenetics 13, 168 (2021). 10.1186/s13148-021-01155-w 34454584 PMC8403420

[47] Wu A. Genome-wide plasma DNA methylation features of metastatic prostate cancer. J Clin Invest 130, 1991–2000 (2020). 10.1172/JCI130887 32149736 PMC7108919

[48] Dillinger T. Identification of tumor tissue-derived DNA methylation biomarkers for the detection and therapy response evaluation of metastatic castration resistant prostate cancer in liquid biopsies. Mol Cancer 21, 7 (2022). 10.1186/s12943-021-01445-0 34980142 PMC8722310

[49] Chen S. The cell-free DNA methylome captures distinctions between localized and metastatic prostate tumors. Nat Commun 13, 6467 (2022). 10.1038/s41467-022-34012-2 36309516 PMC9617856

[50] Feng S., Zhong Z., Wang M. & Jacobsen S. E. Efficient and accurate determination of genome-wide DNA methylation patterns in Arabidopsis thaliana with enzymatic methyl sequencing. Epigenetics Chromatin 13, 42 (2020). 10.1186/s13072-020-00361-9 33028374 PMC7542392

[51] Vaisvila R. Enzymatic methyl sequencing detects DNA methylation at single-base resolution from picograms of DNA. Genome Res 31, 1280–1289 (2021). 10.1101/gr.266551.120 34140313 PMC8256858

[52] Zhao S. G. The DNA methylation landscape of advanced prostate cancer. Nat Genet 52, 778–789 (2020). 10.1038/s41588-020-0648-8 32661416 PMC7454228

